# [Retracted] Downregulated expression levels of USP46 promote the resistance of ovarian cancer to cisplatin and are regulated by PUM2

**DOI:** 10.3892/mmr.2024.13297

**Published:** 2024-07-30

**Authors:** Lei Xu, Bin Zhang, Wenlan Li

Mol Med Rep 23: 263, 2021; DOI: 10.3892/mmr.2021.11902

Following the publication of this paper, it was drawn to the Editor's attention by a concerned reader that certain of the immunohistochemical data shown in Fig. 1D and the flow cytometric data in Fig. 3K were strikingly similar to data appearing in different form in other papers by different authors at different research institutes that were under consideration for publication at around the same time.

Owing to the fact that the contentious data in the above article were already under consideration for publication prior to its submission to *Molecular Medicine Reports*, the Editor has decided that this paper should be retracted from the Journal. The authors were asked for an explanation to account for these concerns, but the Editorial Office did not receive a reply. The Editor apologizes to the readership for any inconvenience caused.

